# Dietary Inflammatory Index and Health Outcomes: An Umbrella Review of Systematic Review and Meta-Analyses of Observational Studies

**DOI:** 10.3389/fnut.2021.647122

**Published:** 2021-05-19

**Authors:** Fang-Hua Liu, Chuan Liu, Ting-Ting Gong, Song Gao, Hui Sun, Yu-Ting Jiang, Jia-Yu Zhang, Meng Zhang, Chang Gao, Xin-Yu Li, Yu-Hong Zhao, Qi-Jun Wu

**Affiliations:** ^1^Department of Clinical Epidemiology, Shengjing Hospital of China Medical University, Shenyang, China; ^2^Clinical Research Center, Shengjing Hospital of China Medical University, Shenyang, China; ^3^Department of Obstetrics and Gynecology, Shengjing Hospital of China Medical University, Shenyang, China

**Keywords:** dietary inflammatory index, health outcomes, meta-analysis, umbrella review, observational studies

## Abstract

**Background and Aims:** The dietary inflammatory index (DII) is associated with non-communicable disease. We conducted an umbrella review to systematically evaluate meta-analyses of observational studies on DII and diverse health outcomes.

**Methods:** We comprehensively searched the PubMed, Web of Science, and Embase databases to identify related systematic reviews and meta-analyses of observational studies. Those investigating the association between DII and a wide range of health outcomes in humans were eligible for inclusion. For each meta-analysis, we estimated the summary effect size by using fixed and random effects models, the 95% confidence interval, and the 95% prediction interval. We assessed heterogeneity, evidence of small-study effects, and excess significance bias.

**Results:** The umbrella review identified 35 meta-analyses assessing associations between DII and various health outcomes: cancer (*n* = 24), mortality (*n* = 4), metabolic (*n* = 4), and other (*n* = 3). The methodological quality was high or moderate. Of the 35 meta-analyses, we observed highly suggestive evidence for harmful associations between digestive tract cancer, colorectal cancer, overall cancer, pharyngeal cancer, UADT cancer, and CVD mortality. Moreover, 11 harmful associations showed suggestive evidence: hormone-dependent cancer, rectal cancer, colon cancer, breast and prostate cancer, gynecological cancer, breast cancer, ovarian cancer, colorectal cancer, prostate cancer, all-cause mortality, and depression.

**Conclusion:** DII is likely to be associated with harmful effects in multiple health outcomes. Robust randomized controlled trials are warranted to understand whether the observed results are causal.

**Systematic Review Registration:** CRD42021218361

## Introduction

The dietary inflammatory index (DII) is a new dietary index developed to reflect the inflammatory potential of diet. It scores an individual's diet on a continuum from anti- to pro-inflammatory. This scoring system is based on the results of scientific publications rather than population means or recommended intakes. The literature includes studies on the relationships among dietary factors—including foods, nutrients, and other bioactive compounds—and six inflammatory biomarkers: C-reactive protein, interleukin (IL)-1b, IL-4, IL-6, IL-10, and tumor necrosis factor-α (1–7). The index distinguishes individuals' diets on the basis of their inflammatory potential on a spectrum from maximally pro-inflammatory components to maximally anti-inflammatory components (1). A higher DII score indicates a more pro-inflammatory diet, whereas a lower DII score indicates a more anti-inflammatory diet. Therefore, the DII offers a valid and readily comparable method for evaluating individuals' dietary inflammatory potential according to the pro- and anti-inflammatory aspects of the overall diet (7). In recent years, the role of DII in relation to non-communicable diseases, including cardiovascular disease (CVD) (8, 9), metabolic syndrome (10), and various types of cancer (11), has been examined in the epidemiological literature. According to the consensus, higher DII scores are deleterious to health. In 2019, a narrative review on the association between DII and non-communicable disease risk was published, describing evidence of relationships between DII and a wide array of cancers, CVD risk and mortality, on the basis of a descriptive presentation of the results of individual studies without quantitative synthesis (12). Although previous studies have examined this topic, a quantitative appraisal of epidemiological credibility is lacking, as are examinations of the potential bias between DII and health-related outcomes and assessments of the most influential outcomes.

In this context, we performed an umbrella review of the evidence through current systematic reviews and meta-analyses of observational studies, to provide an overview of the range and validity of the published relationships between DII and health-related outcomes. We summarized the multiple health outcomes that have been associated with DII in meta-analyses, assessed the diverse bias in these meta-analyses, and identified which of the previously studied associations are supported by the strongest epidemiological evidence.

## Materials and Methods

### Umbrella Review Methods

Umbrella reviews perform in-depth evaluations of the quantitative results of meta-analyses of observational associations and the potential bias in these meta-analyses. Umbrella reviews comprehensively and systematically search and evaluate existing systematic reviews and meta-analyses and can readily be used by decision-makers in healthcare to understand a broad topic field (13).

### Literature Search

We systematically searched PubMed, Embase, and the Web of Science from inception until August 8, 2020, to identify systematic reviews and meta-analyses examining the association between DII and any health outcome. The search terms included DII, dietary inflammatory score, dietary score, inflammatory diet, inflammatory potential of diet, dietary inflammation potential, inflammatory potential intake, anti-inflammatory diet, pro-inflammatory diet, dietary pattern, diet-related inflammation, index-based dietary patterns, or DII, combined with systematic review or meta-analysis (Supplementary Table 1). We also hand-checked the reference lists of eligible systematic reviews and meta-analyses. Potentially eligible articles were retrieved independently by two researchers (F-HL and MZ), and any discrepancies were resolved by a third author (Q-JW).

### Eligibility Criteria

Studies were included if they met the following criteria, established by using the PICOS strategy:

Population: adults.
Intervention/comparison: the DII (including categorical and continuous variables).
Outcomes: health outcomes (e.g., cancer, CVD, mortality, etc.).
Study design: meta-analyses of observational studies (cohort, case–control, or cross-sectional studies).
In addition, if an article performed different meta-analyses on more than one health outcome, each analysis was assessed separately. When two or more meta-analyses were available on the same scientific question, the one including the largest number of studies was selected.

Studies were excluded if they met the following criteria, established by using the PICOS strategy:

Population: non-adults.
Intervention/comparison: not DII.
Outcomes: any other outcome outside of the inclusion criteria.
Study design: systematic reviews of observational studies without quantitative analysis, meta-analyses not reporting comprehensive data (e.g., effect sizes, 95% CIs, and the numbers of cases, controls and total participants), or meta-analyses including primary studies are <3.

### Data Extraction

Two independent authors (F-HL and MZ) extracted data from each eligible meta-analysis, and disagreements were resolved by the third author (Q-JW). First-author name, publication year, journal, number of total studies, study design, method of exposure ascertainment (e.g., food-frequency questionnaire, 24-h recall, etc.), and outcomes were abstracted from each included meta-analysis. From each observational study included in the meta-analysis, we further recorded the name of the first author, publication year, number of cases and controls (case–control study), sample size, measure of exposure (highest vs. lowest category, or dose–response), specific risk estimates, and 95% CIs.

### Data Analysis

For each chosen meta-analysis, we re-calculated the summary effect size estimate, its 95% CIs, and *P*-values by using both fixed- and random-effects models (14, 15). For the summary random effects, we estimated the 95% prediction interval (PI), which accounts for the degree of between-study heterogeneity and indicates the uncertainty for the effect that would be expected in another study examining the same association (16, 17). For the largest study in each meta-analysis, the standard error (SE) of the effect size was calculated, and we examined whether the SE was <0.10. We used the *I*^2^ metric to quantify the heterogeneity between studies. *I*^2^ ranges from 0 to 100% and quantifies the variability in effect estimates that results from heterogeneity other than sampling error (18). When the *I*^2^ exceeded 50% or 75%, the heterogeneity was judged to be large or very large, respectively.

We conducted Egger's regression asymmetry test, proposed by Egger and colleagues (19), to estimate the small-study effects, that is, whether small studies tended to present higher risk estimates than large studies. A *P*-value < 0.10 combined with more conservative effects in larger studies than in random-effects meta-analysis was considered to constitute adequate evidence of small-study effects.

We further evaluated whether the observed number of studies (O) with nominally significant results (positive studies, *P* < 0.05) included in a meta-analysis was larger than the expected number (E), by using the excess statistical significance test (20). We used the effect size of the largest study (with the smallest SE) in each meta-analysis to investigate the power of the component studies by using a non-central t-distribution. A *P*-value < 0.10 (one-sided *P* < 0.05 combined with O > E as early suggested) was considered to indicate excess statistical significance for each meta-analysis (20).

### Assessment of the Quality and Grading of Evidence

The 11-item AMSTAR (21) checklist was used by two independent authors to assess the methodological quality of systematic reviews and meta-analyses. The AMSTAR checklist is a strict, reliable, and valid measurement tool to evaluate systematic reviews and meta-analyses, which includes the quality of the search, analysis, and transparency of meta-analyses. The AMSTAR score is graded as high, moderate, low, or critically low quality.

According to previous umbrella reviews (22, 23), we categorized the evidence from meta-analyses with nominally significant summary results (*P* < 0.05) as convincing, highly suggestive, suggestive, weak, or non-significant associations. We considered convincing associations to have a statistical significance of *P* < 10^−6^ in a random-effects model based on >1,000 cases (or >20,000 participants for continuous outcomes), *P* < 0.05 for the largest component study, a 95% PI excluding the null value, no heterogeneity (*I*^2^ < 50%), and no evidence of small-study effects (*P* > 0.10) or excess significance bias (*P* > 0.10). Highly suggestive evidence had a significance threshold of *P* < 10^−6^, a number of cases >1,000 (or > 20,000 participants for continuous outcomes), and a statistically significant effect observed in the largest study. Suggestive evidence was indicated by >1,000 cases (or >20,000 participants for continuous outcomes) and *P* < 10^−3^ for random effects. Weak evidence was indicated by *P* < 0.05 for a significant association. No association was indicated by a *P*-value not reaching the significance threshold (*P* > 0.05).

Association does not necessarily indicate causation. For associations with convincing or highly suggestive evidence, we performed a sensitivity analysis of only prospective cohort studies to investigate the temporality of the relationship between DII and any health outcome. All analyses were conducted in STATA, version 12.0.

## Results

### Study Selection

**Figure 1:**
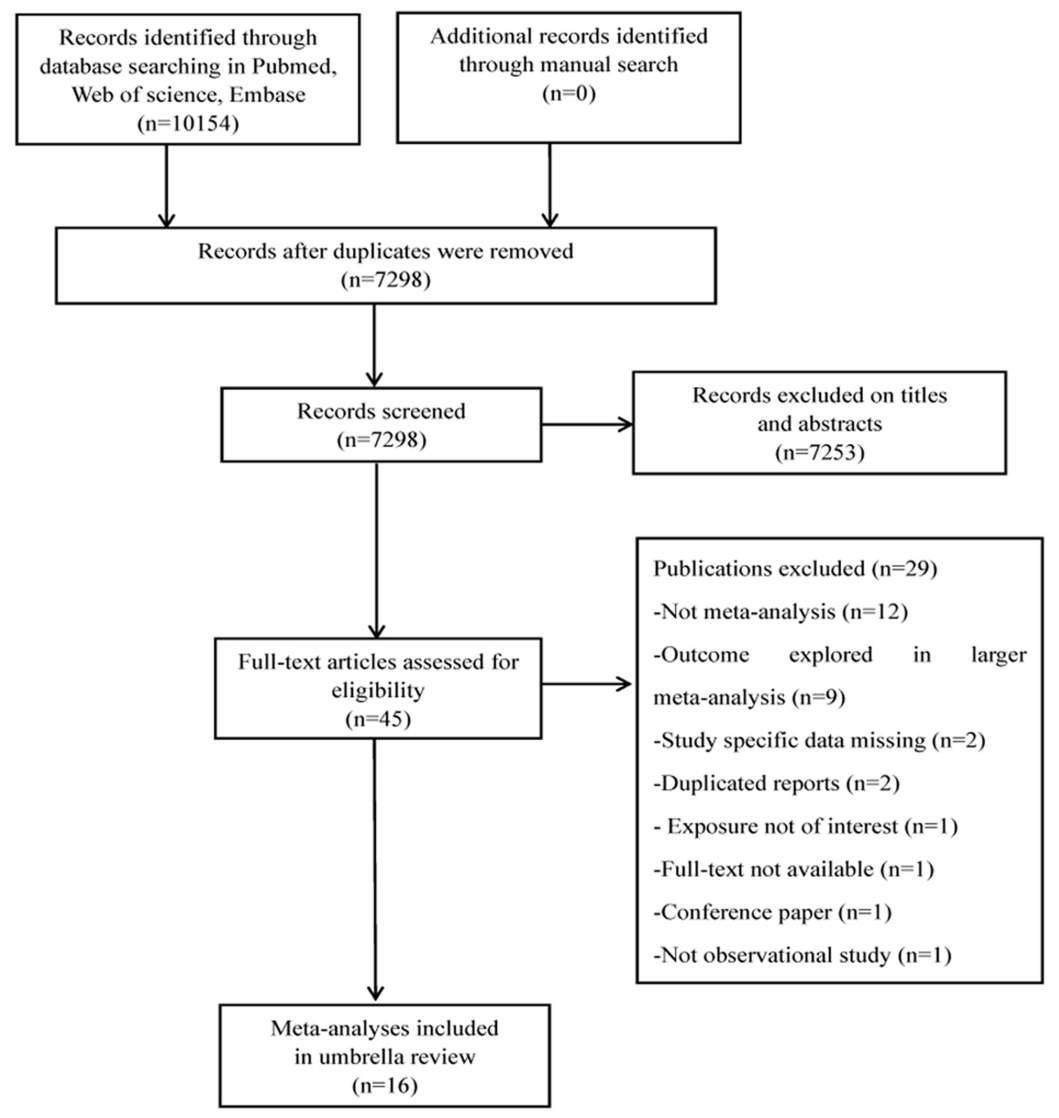
Flowchart of selection of studies for inclusion in umbrella review on DII and health outcomes.

Overall, 10,154 publications were retrieved through the database search, and 45 were deemed eligible. Twenty-nine publications were excluded after full-text screening for different reasons (Figure 1 and Supplementary Table 2). The final selection yielded 16 articles to be included for analysis (9, 24–38).

### Characteristics of Meta-Analyses

Table 1 shows the 35 independent meta-analyses, which included 261 primary studies in 16 articles (9, 24–38). These 16 articles were published between 2018 and 2020. Cohort, case–control, and cross-sectional studies were included in the meta-analyses, and the median number of studies per meta-analysis was seven (range: 3–44). The case numbers exceeded 1,000 in 30 meta-analyses. A wide range of outcomes were investigated: 24 (69%) of 35 meta-analyses studied associations between DII and cancers, including digestive tract cancer, respiratory tract cancer, hormone-dependent cancer, rectal cancer, colon cancer, breast and prostate cancer, gynecological cancers, breast cancer, ovarian cancer, colorectal cancer, prostate cancer, overall cancer, esophageal cancer, lung cancer, oral cavity cancer, pharynx cancer, larynx cancer, upper aerodigestive tract (UADT) cancer, and gastric cancer. Four (11%) of 35 meta-analyses examined mortality and metabolic outcomes. In addition, three meta-analyses investigated associations with CVD risk (*n* = 2) and depression (*n* = 1). Twenty-eight meta-analyses compared the highest vs. lowest DII score, and seven meta-analyses reported health effects associated with a one-unit increase in DII score.

**Table 1:** Characteristics and quantitative synthesis of the eligible meta-analyses of DII for health outcomes.

**Outcomes (reference)**	**First author, year**	**Study design included in meta-analysis**	**No. of studies**	**No. of cases/participants**	**Level of comparison**	**Summary effect size (95% CI)**	
						**Random effects**	**Fixed effects**
**Cancer outcomes**							
Breast and prostate cancer (26)	Moradi S, 2018	Cohort and Case–control study	9	9,972/52,203	Highest vs. lowest	1.70 (1.31–2.22)	1.40 (1.27–1.55)
Breast cancer (30)	Liu, 2019	Cohort and Case–control study	12	30,052/347,147	Highest vs. lowest	1.34 (1.14–1.56)	1.19 (1.14–1.24)
Breast cancer (31)	Jayedi A, 2018	Cohort and Case–control study	7	18,781/225,606	A 1-unit increment	1.04 (1.00–1.08)	1.01 (1.00–1.02)
Colon cancer (25)	Zhang, 2017	Cohort and Case–control study	6	8,210/389,847	Highest vs. lowest	1.37 (1.16–1.62)	1.26 (1.18–1.36)
Colorectal cancer (37)	Moazzen S, 2020	Cohort and Case–control study	11	28,645/1,037,658	Highest vs. lowest	0.66 (0.56–0.78)	0.79 (0.75–0.83)
Colorectal cancer (31)	Jayedi A, 2018	Cohort and Case–control study	9	18,888/878,912	A 1-unit increment	1.06 (1.04–1.09)	1.04 (1.01–1.05)
Digestive tract cancer (24)	Zahedi H, 2020	Cohort and Case–control study	14	15,399/694,894	Highest vs. lowest	1.83 (1.53–2.19)	1.29 (1.23–1.35)
Esophageal cancer (34)	Li, 2018	Cohort and Case–control study	5	891/3,598	Highest vs. lowest	2.81 (2.07–3.82)	2.74 (2.11–3.57)
Gastric cancer (36)	Liang, 2019	Cohort and Case–control study	3	700/2,118	Highest vs. lowest	2.12 (1.41–3.18)	1.95 (1.48–2.57)
Gastric cancer (36)	Liang, 2019	Cohort and Case–control study	3	475/101,835	A 1-unit increment	1.45 (1.04–2.03)	1.24 (1.12–1.38)
Gynecological cancers (30)	Liu, 2019	Cohort and Case–control study	18	33,907/357,095	Highest vs. lowest	1.38 (1.21–1.56)	1.21 (1.16–1.26)
Hormone-dependent cancer (24)	Zahedi H, 2020	Cohort and Case–control study	14	22,234/239,666	Highest vs. lowest	1.22 (1.10–1.34)	1.10 (1.06–1.15)
Larynx cancer (35)	Hua, 2020	Case–control study	3	997/2,805	Highest vs. lowest	2.05 (0.85–4.93)	2.67 (1.95–3.66)
Lung cancer (34)	Li, 2018	Cohort and Case–control study	6	2,162/7,707	Highest vs. lowest	1.56 (1.21–2.01)	1.45 (1.22–1.73)
Oral cavity cancer (35)	Hua, 2020	Case–control study	3	926/3,371	Highest vs. lowest	2.23 (1.73–2.86)	2.23 (1.73–2.86)
Overall cancer (34)	Li, 2018	Cohort and Case–control study	44	48,032/1,298,343	Highest vs. lowest	1.58 (1.45–1.72)	1.37 (1.32–1.42)
Overall cancer (34)	Li, 2018	Cohort and Case–control study	30	31,863/532,225	A 1-unit increment	1.13 (1.09–1.16)	1.08 (1.07–1.10)
Ovarian cancer (30)	Liu, 2019	Cohort and Case–control study	4	3,104/7,982	Highest vs. lowest	1.41 (1.21–1.65)	1.41 (1.21–1.65)
Pharynx cancer (35)	Hua, 2020	Case–control study	4	1,161/9,163	Highest vs. lowest	2.02 (1.54–2.64)	2.00 (1.59–2.51)
Prostate cancer (32)	Zhu, 2019	Cohort and Case–control study	10	5,326/52,873	Highest vs. lowest	1.73 (1.34–2.23)	1.28 (1.17–1.39)
Prostate cancer (32)	Zhu, 2019	Cohort and Case–control study	10	5,326/52,873	A 1-unit increment	1.10 (1.04–1.17)	1.04 (1.02–1.07)
Rectal cancer (25)	Zhang, 2017	Cohort and Case–control study	7	4,679/730,773	Highest vs. lowest	1.44 (1.23–1.69)	1.42 (1.29–1.57)
Respiratory tract cancer (24)	Zahedi H, 2020	Cohort and Case–control study	4	1,261/41,979	Highest vs. lowest	1.80 (1.21–2.67)	1.74 (1.40–2.16)
UADT cancer (35)	Hua, 2020	Case–control study	9	4,394/19,984	Highest vs. lowest	2.27 (1.89–2.73)	1.93 (1.78–2.10)
**Mortality**							
All-cause mortality (29)	Namazi N, 2018	Cohort and Cross-sectional study	6	32,677/107,306	Highest vs. lowest	1.21 (1.09–1.35)	1.13 (1.09–1.18)
Cancer mortality (24)	Zahedi H, 2020	Cohort and Case–control study	11	9,506/229,115	Highest vs. lowest	1.23 (1.07–1.42)	1.14 (1.07–1.22)
CVD mortality (27)	Ji, 2020	Cohort study	10	32,319/385,765	Highest vs. lowest	1.31 (1.19–1.44)	1.21 (1.16–1.27)
CVD mortality (9)	Shivappa N, 2017	Cohort and Cross-sectional study	6	11,094/93,866	A 1-unit increment	1.09 (1.03–1.15)	1.06 (1.04–1.08)
**Metabolic outcomes**							
Central obesity (38)	Farhangi MA, 2020	Cross-sectional study	10	6,904/25,435	Highest vs. lowest	1.16 (0.95–1.43)	1.06 (0.96–1.17)
Hypertension (33)	Farhangi MA, 2019	Cross-sectional study	12	20,126/44,102	Highest vs. lowest	1.13 (1.01–1.27)	1.15 (1.08–1.23)
Hyperglycemia (33)	Farhangi MA, 2019	Cross-sectional study	9	5,365/10,715	Highest vs. lowest	1.13 (0.95–1.35)	1.05 (0.95–1.16)
Metabolic syndrome (29)	Namazi N, 2018	Cohort and Cross-sectional study	5	2,242/15,161	Highest vs. lowest	1.01 (0.82–1.24)	1.01 (0.87–1.18)
**Other outcomes**							
CVD (27)	Ji, 2020	Cohort study	6	1,310/43,385	Highest vs. lowest	1.41 (1.12–1.78)	1.35 (1.13–1.61)
CVD (9)	Shivappa N, 2017	Cohort and Cross-sectional study	4	2,420/49,446	A 1-unit increment	1.08 (1.00–1.16)	1.03 (1.01–1.05)
Depression (28)	Wang, 2018	Cohort and Cross-sectional study	6	4,864/49,584	Highest vs. lowest	1.23 (1.12–1.35)	1.23 (1.12–1.35)

*CVD, cardiovascular disease; DII, dietary inflammatory index; UADT, upper aerodigestive tract*.

### Summary Effect Size

Of the 35 meta-analyses, the summary random effects estimates were significant at *P* < 0.05 in 31 (89%) meta-analyses, whereas the summary fixed effects estimates were significant in 32 (91%) meta-analyses. When *P* < 0.001 was taken as a threshold for significance, 21 (60%) and 29 (83%) meta-analyses generated significant summary results according to the random and fixed effects models, respectively. However, when a more stringent threshold of significance (*P* < 1 × 10^−6^) was applied, the summary random effects estimates were significant in nine (26%) meta-analyses, and the summary fixed effects estimates were significant in 20 (57%) meta-analyses (Supplementary Table 3). All nine meta-analyses with strongly significant significance under the random effects model reported an increased risk. Of these, seven associations (digestive tract cancer, overall cancer, esophageal cancer, oral cavity cancer, pharynx cancer, UADT cancer, and CVD mortality) were studied by comparison of the highest vs. lowest DII score, and two associations (colorectal cancer and overall cancer) were investigated for dose–response relationships. The magnitude of the observed summary random effect estimates ranged from 0.66 to 2.81 (Supplementary Figure 1).

The effect of the largest study included in each meta-analysis is reported in our analysis (Supplementary Table 3). Twenty-one (60%) of 35 meta-analyses reported that the largest study effect was nominally statistically significant, with a *P*-value < 0.05, and a more conservative effect than the summary random effects was observed in 25 (71%) of 35 meta-analyses.

### Heterogeneity and Prediction Intervals

Of the 35 meta-analyses, 9 (26%) showed low heterogeneity (*I*^2^ < 50%), 13 (37%) meta-analyses showed high heterogeneity (*I*^2^ = 50–75%), and 13 (37%) showed very high heterogeneity (*I*^2^ > 75%) (Supplementary Table 4). When 95% PIs were evaluated, we found that seven meta-analyses (ovarian cancer, colorectal cancer, overall cancer, esophageal cancer, pharynx cancer, UADT cancer, and depression) excluded the null value (Supplementary Table 4).

### Small-Study Effects and Excess Significance Bias

Evidence of small-study effects was observed in 19 (54%) of 35 meta-analyses on the basis of Egger's test (Supplementary Table 4). When taking the largest study estimate as the plausible effect size, we found that 19 (54%) of 35 meta-analyses showed evidence of excess significance (i.e., compared with larger studies, smaller studies tended to show substantially larger estimates of effect size) (Supplementary Table 4).

### Methodological Quality of the Meta-Analyses

Among the 16 articles included in our umbrella review, only two (13%) were rated as high quality (≥8 points) and 14 (87%) were defined as moderate quality (4–7 points), according to the AMSTAR criteria (Supplementary Figure 2). In summary, the common flaws were that gray literature was not considered in the literature search, the list of excluded studies was not presented, and the influence of the quality of the included studies was not discussed.

### Evidence Grading

**Figure 2:**
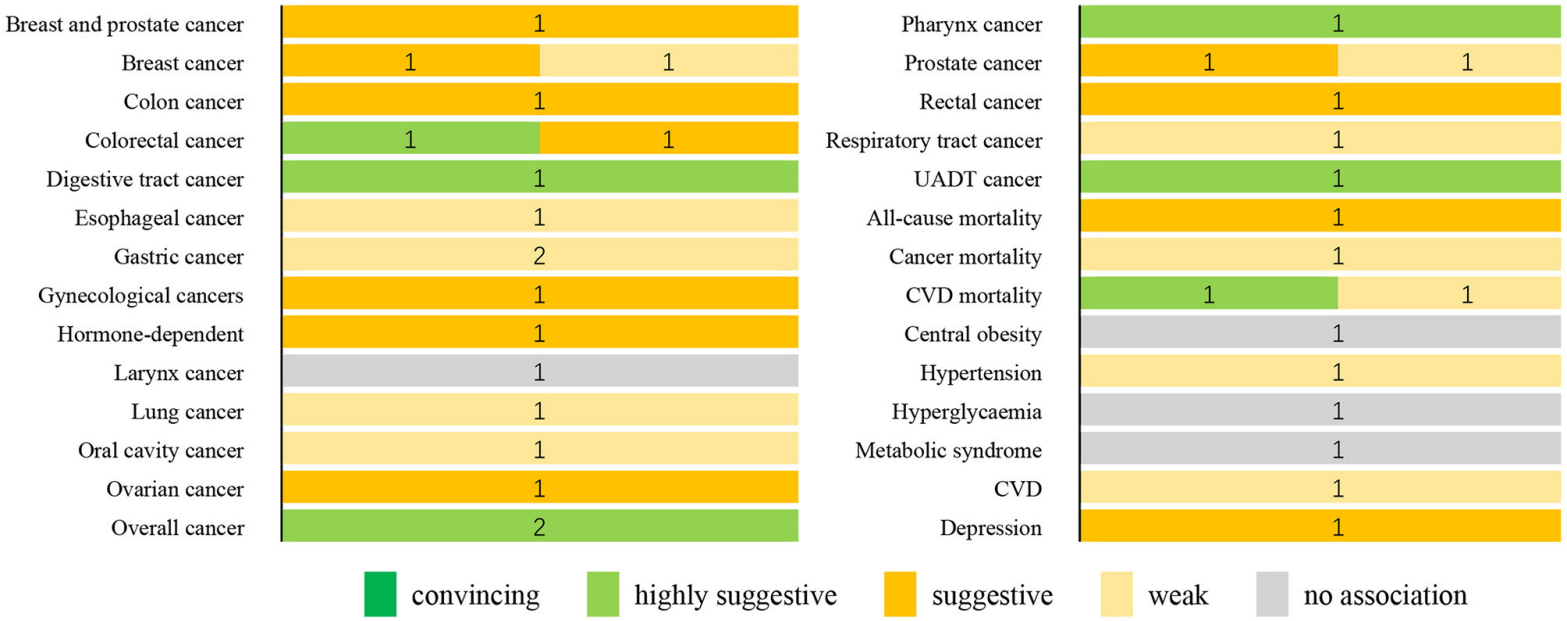
Summary of the strength of evidence for the evaluated health outcomes. Numbers indicate the number of meta-analyses with convincing, highly suggestive, weak, or no association for each outcome. CVD, cardiovascular disease; UADT, upper aerodigestive tract.

Figure 2 summarizes the strength of evidence of the meta-analyses that evaluated the DII on health outcomes. On the basis of the predefined methodological criteria, no association presented convincing evidence, whereas seven associations presented highly suggestive evidence. These associations are as follows: the effect of DII on the risk of digestive tract cancer (highest vs. lowest), colorectal cancer (a one-unit increment), overall cancer (highest vs. lowest and a one-unit increment), pharynx cancer (highest vs. lowest), UADT cancer (highest vs. lowest), and CVD mortality (highest vs. lowest). Eleven associations were supported by suggestive evidence: hormone-dependent cancer, rectal cancer, colon cancer, breast and prostate cancer, gynecological cancer, breast cancer, ovarian cancer, colorectal cancer, prostate cancer, all-cause mortality, and depression. Moreover, 13 associations were supported by weak evidence. Finally, for the other four associations, a non-significant result was found.

We performed a sensitivity analysis for associations supported by convincing and highly suggestive evidence, which was limited to meta-analyses of prospective cohort studies. Of the five associations included, three further associations met the criteria for highly suggestive evidence: the effect of DII on colorectal cancer (DII as a continuous variable), overall cancer, and CVD mortality. Two associations between DII and digestive tract cancer and overall cancer (DII as continuous variable) were downgraded to suggestive evidence (Supplementary Table 6).

## Discussion

### Principal Findings

The present study is the first umbrella review to quantitatively estimate the existing evidence of the associations between DII and multiple health outcomes, including cancer, mortality, metabolic, and other outcomes (i.e., CVD and depression). We provided a comprehensive overview of the current meta-analyses of observational studies and examined the methodological quality of the included meta-analyses and the quality of evidence for all these associations.

The umbrella review comprised 16 published meta-analyses on a total population of more than 5.6 million participants. The methodological quality of the included meta-analyses was high/moderate. Highly suggestive evidence was observed for seven outcome variables, including digestive tract cancer, colorectal cancer, overall cancer, pharynx cancer, UADT cancer, and CVD mortality. Eleven outcomes associated with higher DII scores presented suggestive evidence. Additionally, 11 associations were supported by weak evidence.

### Interpretation in Light of Evidence

The most notable associations identified in our umbrella review were those between DII and diverse cancers; harmful associations of DII with digestive tract cancer, colorectal cancer, overall cancer, pharynx cancer, and UADT cancer were observed. Our results were in accordance with those of prior studies. A population-based multi case–control study in Spain (39) showed that a one-unit DII score increment is associated with increased colorectal cancer risk. A meta-analysis of prospective studies (10), including more than 28,000 participants, reported an association between DII and elevated risk of cancer, although the dose–response association requires further investigation. However, the selected meta-analyses examining the above associations have some limitations, such as the substantial heterogeneity, the 95% PI containing the null value, and the presence of small-study effects and excess significance bias. Of particular interest is the relationship between DII and pharynx cancer. Our research included excess significance bias, whereas there was no evidence of the 95% PI including the null value or small-study effects. Compared with a previous umbrella review that explored the association between single risk factors and health-related outcomes (40, 41), the association between DII and pharynx cancer risk did not meet only one of our criteria for convincing evidence, thus suggesting that the present bias in the study of the association between DII and pharynx cancer risk might be relatively modest. The mechanisms underlying the harmful role of diet-related inflammation in increasing the risk of cancer have been widely explored. Systemic inflammation may cause an increase in insulin resistance, which may in turn affect the risk of digestive-tract cancers (42, 43). In addition, pro-inflammatory diets, such as a Western diet, may contribute to overweight, high BMI, and obesity (44). Previous studies have suggested that high BMI or obesity is correlated with increased cancer risk (45, 46). Furthermore, interactions between diet and the environment are considered to be associated with epigenetic changes and cancer (47, 48).

We found highly suggestive evidence that higher DII scores are associated with a higher risk of CVD mortality. Our results are in line with those from a published meta-analysis comprising 42,000 participants and 1,000 CVD cases, in which a higher DII score was found to increase the risk of CVD mortality (49). However, this result must be interpreted with caution, mainly because of the high heterogeneity, the 95% PI including the null value, and the evidence of small-study effects and excess significance bias. Further studies are therefore needed. The effect of a pro-inflammatory diet on insulin resistance may explain the observed relationship between DII with increased CVD mortality risk (50, 51). Insulin resistance is a common pathway for both cancer (52) and CVD (53). Moreover, the DII has been confirmed to be associated with higher levels of high-sensitivity C-reactive protein (hs-CRP) (a serum inflammatory marker) (54). High levels of hs-CRP predict the risk of CVD mortality. If hs-CRP is not controlled, progressive disease and death may result.

Furthermore, we observed suggestive evidence of a positive association between DII and depression. This association did not meet our criteria for convincing evidence, mainly because of the relatively low statistical significance. A systematic review examining DII and the risk of depression, published in 2019, has also reported that higher DII is associated with a higher risk of depression (55). Pro-inflammatory diets may increase inflammatory response system activation. Previous studies have also shown that in depressed people, inflammation is increased (56, 57), and the availability of brain-derived neurotrophic factor is decreased (58). Moreover, possible mechanisms underlying the effects of diet on depression include oxidative stress, the hypothalamic–pituitary–adrenal axis, and tryptophan depletion (59).

### Strengths and Limitations

This umbrella review is the first to provide a comprehensive critical appraisal of published systematic reviews and meta-analyses on all health outcomes for DII. In addition, we searched three databases through a rigorous strategy, and two authors independently extracted the information. Moreover, we used the AMSTAR criteria to assess the methodological quality of selected in our umbrella review, and all investigated meta-analyses achieved a moderate-to-high quality score, thereby suggesting that current meta-analyses evaluating the effects of the DII on health outcomes partially or almost fully complied with standards of methodological quality. We used the criteria of evidence grading to evaluate the evidence categorization.

Nevertheless, some limitations of our study must be discussed. First, assessment criteria for credibility, which were based on established tools for observational evidence, were used in our umbrella review. Although none of the components of the assessment criteria presented definite evidence of absence of reliability, they together indicated the susceptibility of the results to uncertainty and bias. To account for the discrepancies in the populations, study designs, or other characteristics of the studies included in the meta-analyses, we used an *I*^2^ < 50% as a criterion for convincing evidence, to assign the best grade of evidence to only robust associations without heterogeneity. Unfortunately, many of the meta-analyses included in our umbrella review showed high or very high heterogeneity. Second, fewer than 10 original studies were included in most (74%) of the meta-analyses in our study, thus potentially decreasing the power of Egger's tests and excess significance tests (22). Moreover, we used the random effects model proposed by DerSimonian and Laird (17) to calculate the adjusted summary effect size and corresponding 95% CIs, to ensure comparability with prior meta-analyses. However, a better approach to reflect the uncertainty in the variance between studies is recommended for future studies, such as the Hartung–Knapp approach (60), which shows the uncertainty of variance by wider CIs. Third, our work depended on prior meta-analyses, which might have missed some individual studies. However, this aspect is unlikely to have affected our findings, because the meta-analyses included in our study were those that included the largest number of research studies. Fourth, the results' validity depended on the quality of the data extracted from the meta-analyses included in the present umbrella review; those studies had the common limitations of observational studies, such as self-reported dietary information, misclassification, recall bias, or confounding bias. Fifth, few systematic reviews and meta-analyses included in our umbrella review performed analysis for confounding factors (Supplementary Table 7). However, most studies included in the meta-analyses in this umbrella review did adjust for potential confounding factors, including age, sex, and race.

## Conclusions

In summary, highly suggestive evidence exists for associations between DII and digestive tract cancer, colorectal cancer, cancer, pharynx cancer, UADT cancer, and CVD mortality. Although DII may be associated with an increased risk of other health outcomes, their relationships are uncertain according to our study. In the future, better designed studies are necessary to generate definite conclusions, and the association between inflammatory markers and health outcomes in a given population must be investigated.

## Transparency

The corresponding author affirms that the manuscript is an honest, accurate, and transparent account of the study being reported; that no important aspects of the study have been omitted; and that any discrepancies from the study as planned have been explained.

## Data Availability Statement

The data supporting the conclusions of this article can be directed to the corresponding author/s.

## Author Contributions

Q-JW, SG, and Y-HZ conceived the study. F-HL, SG, and T-TG contributed to the design. F-HL and MZ conducted the literature search, literature screening, and extracted the data. HS, Y-TJ, and J-YZ performed the quality assessment. X-YL and CG performed the statistical analysis. F-HL and T-TG wrote the first draft of the paper. CL revised the manuscript for important content. SG is the guarantor. The corresponding author attests that all listed authors meet authorship criteria and that no others meeting the criteria were omitted. All authors interpreted the data, read the manuscript, and approved the final vision.

## Conflict of Interest

The authors declare that the research was conducted in the absence of any commercial or financial relationships that could be construed as a potential conflict of interest.
