# Supplementary material for: Dietary Inflammatory Index and Health Outcomes: An Umbrella Review of Systematic Review and Meta-Analyses of Observational Studies
Source: Front Nutr. 2021 May 19;8:647122. doi: 10.3389/fnut.2021.647122 (PMC8169973; doi:10.3389/fnut.2021.647122)
Supplement: Supplementary file 1 [file Data_Sheet_1.doc]

**Supplementary materials**

**Dietary inflammatory index and health outcomes: an umbrella review of systematic and meta-analyses of observational studies**

**Supplementary Table S1. Search strategy used in the umbrella review**

| Literature search strategy in PubMed |
| --- |
| 1: dietary inflammatory index OR dietary inflammatory score OR dietary score OR inflammatory diet OR inflammatory potential of diet OR dietary inflammation potential OR inflammatory potential intake OR anti-inflammatory diet OR pro-inflammatory diet OR dietary pattern OR diet-related inflammation OR index-based dietary patterns OR DII |
| 2: systematic review OR meta-analysis |
| 3: 1 AND 2 |
| Literature search strategy in Web of Science |
| 1: Search TS = (dietary inflammatory index OR dietary inflammatory score OR dietary score OR inflammatory diet OR inflammatory potential of diet OR dietary inflammation potential OR inflammatory potential intake OR anti-inflammatory diet OR pro-inflammatory diet OR dietary pattern OR diet-related inflammation OR index-based dietary patterns OR DII) |
| 2: Search TS = (systematic review OR meta-analysis) |
| 3: 1 AND 2 |
| Literature search strategy in EMBASE |
| 1: dietary inflammatory index OR dietary inflammatory score OR dietary score OR inflammatory diet OR inflammatory potential of diet OR dietary inflammation potential OR inflammatory potential intake OR anti-inflammatory diet OR pro-inflammatory diet OR dietary pattern OR diet-related inflammation OR index-based dietary patterns OR DII |
| 2: ‘systematic review’ OR ‘meta-analysis’ |
| 3: 1 AND 2 |

**Supplementary Table S2. References of studies excluded in the umbrella review**

| **Not meta-analysis (N=12)** |
| --- |

1. Steffen LM, Hootman KC. A Posteriori Data-Derived Dietary Patterns and Incident Coronary Heart Disease: Making Sense of Inconsistent Findings. Current Nutrition Reports 2016, 5(3): 168-179.

2. Pan P, Yu J, Wang L. Diet and colon: what matters? CURR OPIN GASTROEN 2019, 35(2): 101-106.

3. Kheirouri S, Alizadeh M. Dietary Inflammatory Potential and the Risk of Incident Depression in Adults: A Systematic Review. ADV NUTR 2019, 10(1): 9-18.

4. Kheirouri S, Alizadeh M. Dietary Inflammatory Potential and the Risk of Neurodegenerative Diseases in Adults. EPIDEMIOL REV 2019, 41(1): 109-120.

5. Suhett LG, Hermsdorff H, Cota BC, Ribeiro S, Shivappa N, Hébert JR, Franceschini S, de Novaes JF. Dietary inflammatory potential, cardiometabolic risk and inflammation in children and adolescents: a systematic review. Crit Rev Food Sci Nutr 2020: 1-10.

6. Chen X, Maguire B, Brodaty H, O'Leary F. Dietary Patterns and Cognitive Health in Older Adults: A Systematic Review. J ALZHEIMERS DIS 2019, 67(2): 583-619.

7. Zheng J, Guinter MA, Merchant AT, Wirth MD, Zhang J, Stolzenberg-Solomon RZ, Steck SE. Dietary patterns and risk of pancreatic cancer: a systematic review. NUTR REV 2017, 75(11): 883-908.

8. Steck SE, Guinter M, Zheng J, Thomson CA. Index-based dietary patterns and colorectal cancer risk: a systematic review. ADV NUTR 2015, 6(6): 763-773.

9. Terranova CO, Protani MM, Reeves MM. Overall Dietary Intake and Prognosis after Breast Cancer: A Systematic Review. NUTR CANCER 2018, 70(2): 153-163.

10. O'Connor JP, Milledge KL, O'Leary F, Cumming R, Eberhard J, Hirani V. Poor dietary intake of nutrients and food groups are associated with increased risk of periodontal disease among community-dwelling older adults: a systematic literature review. NUTR REV 2020, 78(2): 175-188.

11. Ruiz-Canela M, Bes-Rastrollo M, Martinez-Gonzalez MA. The Role of Dietary Inflammatory Index in Cardiovascular Disease, Metabolic Syndrome and Mortality. INT J MOL SCI 2016, 17(UNSP 12658).

12. Fernández-Villa T, Álvarez-Álvarez L, Rubín-García M, Obón-Santacana M, Moreno V. The role of dietary patterns in colorectal cancer: a 2019 update. Expert Rev Gastroenterol Hepatol 2020, 14(4): 281-290.

| **Outcome explored in larger meta-analysis (N=9)** |
| --- |

13. Mohseni R, Abbasi S, Mohseni F, Rahimi F, Alizadeh S. Association between Dietary Inflammatory Index and the Risk of Prostate Cancer: A Meta-Analysis. NUTR CANCER 2019, 71(3): 359-366.

14. Namazi N, Larijani B, Azadbakht L. Association between the dietary inflammatory index and the incidence of cancer: a systematic review and meta-analysis of prospective studies. PUBLIC HEALTH 2018, 164: 148-156.

15. Shivappa N, Godos J, Hébert JR, Wirth MD, Piuri G, Speciani AF, Grosso G. Dietary Inflammatory Index and Colorectal Cancer Risk-A Meta-Analysis. NUTRIENTS 2017, 9(9).

16. Zahedi H, Djalalinia S, Sadeghi O, Asayesh H, Noroozi M, Gorabi AM, Mohammadi R, Qorbani M. Dietary Inflammatory Potential Score and Risk of Breast Cancer: Systematic Review and Meta-analysis. CLIN BREAST CANCER 2018, 18(4): e561-e570.

17. Du S, Li Y, Su Z, Shi X, Johnson NL, Li P, Zhang Y, Zhang Q, Wen L, Li K, Chen Y, Zhang X, Fei Y, Ding X. Index-based dietary patterns in relation to gastric cancer risk: a systematic review and meta-analysis. Br J Nutr 2020, 123(9): 964-974.

18. Zhong X, Guo L, Zhang L, Li Y, He R, Cheng G. Inflammatory potential of diet and risk of cardiovascular disease or mortality: A meta-analysis. Sci Rep 2017, 7(1): 6367.

19. Wang L, Liu C, Zhou C, Zhuang J, Tang S, Yu J, Tian J, Feng F, Liu L, Zhang T, Sun C. Meta-analysis of the association between the dietary inflammatory index (DII) and breast cancer risk. EUR J CLIN NUTR 2019, 73(4): 509-517.

20.Fan Y, Jin X, Man C, Gao Z, Wang X. Meta-analysis of the association between the inflammatory potential of diet and colorectal cancer risk. Oncotarget 2017, 8(35): 59592-59600.

21.Lu DL, Ren ZJ, Zhang Q, Ren PW, Yang B, Liu LR, Dong Q. Meta-analysis of the association between the inflammatory potential of diet and urologic cancer risk. PLOS ONE 2018, 13(10): e204845.

| **Study specific data missing (N=2)** |
| --- |

22. Zhu J, Ling Y, Mi S, Chen H, Fan J, Cai S, Fan C, Shen Q, Li Y. Association between dietary inflammatory index and upper aerodigestive tract cancer risk: A systematic review and dose-response meta-analysis. ORAL ONCOL 2020, 103: 104587.

23.Fang Y, Zhu J, Fan J, Sun L, Cai S, Fan C, Zhong Y, Li Y. Dietary Inflammatory Index in relation to bone mineral density, osteoporosis risk and fracture risk: a systematic review and meta-analysis. Osteoporos Int 2020.

| **Duplicated reports (N=2)** |
| --- |

24. Wang J, Zhou Y, Chen K, Jing Y, He J, Sun H, Hu X. Dietary inflammatory index and depression: a meta-analysis. PUBLIC HEALTH NUTR 2019, 22(4): 654-660.

25. Zheng J, Guinter M, Merchant A, Wirth M, Zhang J, Stolzenberg-Solomon R, Steck S. Dietary patterns and risk of pancreatic cancer: A systematic review. FASEB J 2017, 31(1).

| **Exposure not of interest (N=1)** |
| --- |

26. Tolkien K, Bradburn S, Murgatroyd C. An anti-inflammatory diet as a potential intervention for depressive disorders: A systematic review and meta-analysis. CLIN NUTR 2019, 38(5): 2045-2052.

| **Full-text not available (N=1)** |
| --- |

27. Molendijk M, Molero P, Ortuño SF, Van der Does W, Angel MM. Diet quality and depression risk: A systematic review and dose-response meta-analysis of prospective studies. J Affect Disord 2018, 226: 346-354.

| **Conference paper (N=1)** |
| --- |

28. Nicolaou M, Vermeulen E, Elstgeest L, Knuppel A, Colpo M, Schoenaker D, Gibson-Smith D, Pot G, Brouwer I, Visser M. A META-ANALYSIS OF THE ROLE OF A PRIORI DIETARY INDICES IN DEPRESSION AMONG 7 COHORTS; THE MOODFOOD PROJECT. ANN NUTR METAB 2017, 712: 985.

| **Not observational study (N=1)** |
| --- |

29. Shivappa N, Hebert JR, Kivimaki M, Akbaraly T. Alternative Healthy Eating Index 2010, Dietary inflammatory index and risk of mortality: Results from the Whitehall II cohort study and meta-analysis of previous DII and mortality studies (vol 118, pg 210, 2017). BRIT J NUTR 2017, 118(8): 639.

**Supplementary Table S3. Summary effect size for the association of DII for health outcomes.**

| Outcomes (reference) | Summary relative risk (95% CI) | | | Random  *p* value † | Fixed  *p* value ‡ |
| --- | --- | --- | --- | --- | --- |
|  | Random effects | Fixed effects | Largest study* |  |  |
| **Cancer outcomes** | | | | | |
| Breast and prostate cancer(26) | 1.70 (1.31-2.22) | 1.40 (1.27-1.55) | 1.08 (0.86-1.17) | 8.03×10^-5^ | 2.76×10^-11^ |
| Breast cancer(30) | 1.34 (1.14-1.56) | 1.19 (1.14-1.24) | 0.99 (0.91-1.07) | 2.79×10^-4^ | 1.06×10^-14^ |
| Breast cancer(31) | 1.04 (1.00-1.08) | 1.01 (1.00-1.02) | 1.00 (0.99-1.02) | 0.032 | 0.02 |
| Colon cancer(25) | 1.37 (1.16-1.62) | 1.26 (1.18-1.36) | 1.20 (1.09-1.33) | 2.54×10^-4^ | 1.58×10^-10^ |
| Colorectal cancer (37) | 0.66 (0.56-0.78) | 0.79 (0.75-0.83) | 1.01 (0.93-1.10) | 1.62×10^-6^ | 7.53×10^-24^ |
| Colorectal cancer(31) | 1.06 (1.04-1.09) | 1.04 (1.01-1.05) | 1.04 (1.03-1.05) | 1.05×10^-9^ | 2.22×10^-39^ |
| Digestive tract cancer(24) | 1.83 (1.53-2.19) | 1.29 (1.23-1.35) | 1.08 (1.01-1.15) | 3.32×10^-11^ | 8.02×10^-30^ |
| Esophageal cancer(34) | 2.81 (2.07-3.82) | 2.74 (2.11-3.57) | 2.42 (1.57-3.73) | 4.35×10^-11^ | 4.54×10^-14^ |
| Gastric cancer(36) | 2.12 (1.41-3.18) | 1.95 (1.48-2.57) | 1.63 (1.15-2.29) | 2.93×10^-4^ | 1.98×10^-6^ |
| Gastric cancer(36) | 1.45 (1.04-2.03) | 1.24 (1.12-1.38) | 1.19 (1.06-1.34) | 0.029 | 2.22×10^-5^ |
| Gynecological cancers(30) | 1.38 (1.21-1.56) | 1.21 (1.16-1.26) | 0.99 (0.91-1.07) | 1.25×10^-6^ | 5.57×10^-19^ |
| Hormone-dependent cancer(24) | 1.22 (1.10-1.34) | 1.10 (1.06-1.15) | 0.99 (0.91-1.07) | 1.38×10^-4^ | 9.68×10^-6^ |
| Larynx cancer(35) | 2.05 (0.85-4.93) | 2.67 (1.95-3.66) | 3.30 (2.06-5.28) | 0.111 | 9.82×10^-10^ |
| Lung cancer(34) | 1.56 (1.21-2.01) | 1.45 (1.22-1.73) | 1.33 (1.01-1.76) | 0.001 | 3.41×10^-5^ |
| Oral cavity cancer(35) | 2.23 (1.73-2.86) | 2.23 (1.73-2.86) | 2.08 (1.47-2.93) | 3.72×10^-10^ | 3.72×10^-10^ |
| Overall cancer(34) | 1.58 (1.45-1.72) | 1.37 (1.32-1.42) | 1.21 (1.11-1.32) | 1.09×10^-25^ | 2.17×10^-65^ |
| Overall cancer(34) | 1.13 (1.09-1.16) | 1.08 (1.07-1.10) | 1.04 (1.02-1.06) | 7.62×10^-16^ | 4.24×10^-44^ |
| Ovarian cancer(30) | 1.41 (1.21-1.65) | 1.41 (1.21-1.65) | 1.31 (1.06-1.62) | 8.57×10^-6^ | 8.57×10^-6^ |
| Pharynx cancer(35) | 2.02 (1.54-2.64) | 2.00 (1.59-2.51) | 1.64 (1.06-2.55) | 2.81×10^-7^ | 2.79×10^-9^ |
| Prostate cancer(32) | 1.73 (1.34-2.23) | 1.28 (1.17-1.39) | 1.07 (0.95-1.20) | 2.35×10^-5^ | 1.74×10^-8^ |
| Prostate cancer(32) | 1.10 (1.04-1.17) | 1.04 (1.02-1.07) | 1.02 (0.99-1.04) | 0.002 | 1.96×10^-5^ |
| Rectal cancer(25) | 1.44 (1.23-1.69) | 1.42 (1.29-1.57) | 1.45 (1.22-1.73) | 7.65×10^-6^ | 1.80×10^-12^ |
| Respiratory tract cancer(24) | 1.80 (1.21-2.67) | 1.74 (1.40-2.16) | 1.31(0.91-1.89) | 0.004 | 6.36×10^-7^ |
| UADT cancer(35) | 2.27 (1.89-2.73) | 1.93 (1.78-2.10) | 1.71 (1.54-1.90) | 1.13×10^-18^ | 1.67×10^-55^ |
| **Mortality** | | | | | |
| All-cause mortality(29) | 1.21 (1.09-1.35) | 1.13 (1.09-1.18) | 1.08 (1.03-1.13) | 4.69×10^-4^ | 7.54×10^-10^ |
| Cancer mortality(24) | 1.23 (1.07-1.42) | 1.14 (1.07-1.22) | 1.08 (0.99-1.18) | 0.004 | 1.79×10^-4^ |
| CVD mortality(27) | 1.31 (1.19-1.44) | 1.21 (1.16-1.27) | 1.09 (1.01-1.18) | 4.09×10^-8^ | 1.05×10^-18^ |
| CVD mortality(9) | 1.09 (1.03-1.15) | 1.06 (1.04-1.08) | 1.04 (1.01-1.07) | 0.002 | 1.25×10^-7^ |
| **Metabolic outcomes** | | | | | |
| Central obesity(38) | 1.16 (0.95-1.43) | 1.06 (0.96-1.17) | 0.93(0.81-1.06) | 0.154 | 0.232 |
| Hypertension(33) | 1.13 (1.01-1.27) | 1.15 (1.08-1.23) | 1.25(1.07-1.45) | 0.028 | 2.56×10^-5^ |
| Hyperglycaemia(33) | 1.13 (0.95-1.35) | 1.05 (0.95-1.16) | 0.95(0.77-1.18) | 0.173 | 0.299 |
| Metabolic syndrome(29) | 1.01 (0.82-1.24) | 1.01 (0.87-1.18) | 0.96(0.77-1.19) | 0.953 | 0.905 |
| **Other outcomes** |  |  |  |  |  |
| CVD(27) | 1.41 (1.12-1.78) | 1.35 (1.13-1.61) | 1.03(0.76-1.42) | 0.004 | 0.001 |
| CVD(9) | 1.08 (1.00-1.16) | 1.03 (1.01-1.05) | 1.02(1.00-1.04) | 0.045 | 0.005 |
| Depression(28) | 1.23 (1.12-1.35) | 1.23 (1.12-1.35) | 1.23(1.05-1.45) | 1.70×10^-5^ | 1.70×10^-5^ |

Abbreviation：CVD: cardiovascular disease; DII: dietary inflammatory index; UADT: upper aerodigestive tract.

* Relative risk and 95% confidence interval of largest study (smallest SE) in each meta-analysis.

† *P* value of summary random effects estimate.

‡ *P* value of summary fixed effects estimate.

All statistical tests were two-sided.

**Supplementary Table S4. Level of evidence for the association of DII for health outcomes.**

| Outcomes (reference) | Features used for classification of level of evidence | | | | | | | | Evidence class |
| --- | --- | --- | --- | --- | --- | --- | --- | --- | --- |
|  | Significance threshold reached* | I^2^ (95% CI) | 95% prediction interval | Egger’s p value | Excess significance§ | | Largest study significant | Small-study effect/excess significant bias |  |
|  |  |  |  |  | O/E# | p value¶ |  |  |  |
| **Cancer outcomes** | | | | | | | | | |
| Breast and prostate cancer(26) | < 0.001 but > 10^-6^ | 78.3% (59%-89%) | 0.75-3.85 | 0.04 | 7/4.67 | 0.12 | No | Yes/No | Suggestive |
| Breast cancer(30) | < 0.001 but > 10^-6^ | 89.9% (84%-93%) | 0.76-2.33 | 0.09 | 8/6.80 | 0.49 | No | Yes/No | Suggestive |
| Breast cancer(31)** | < 0.05 but > 0.001 | 87.1% (76%-93%) | 0.92-1.18 | 0.10 | 3/2.82 | 0.89 | No | No/No | Weak |
| Colon cancer(25) | < 0.001 but > 10^-6^ | 74.9% (43%-89%) | 0.80-2.33 | 0.12 | 5/2.21 | 0.02 | Yes | No/Yes | Suggestive |
| Colorectal cancer(37) | < 0.001 but > 10^-6^ | 90.3% (85%-94%) | 0.36-1.20 | 0.03 | 9/8.06 | 0.52 | No | Yes/No | Suggestive |
| Colorectal cancer(31)** | < 10^-6^ | 75.7% (53%-87%) | 1.01-1.12 | 0.01 | 9/3.24 | 6.43×10^-5^ | Yes | Yes/Yes | Highly suggestive |
| Digestive tract cancer(24) | < 10^-6^ | 89.4% (84%-93%) | 0.97-3.46 | 0.00 | 14/10.49 | 0.03 | Yes | Yes/Yes | Highly suggestive |
| Esophageal cancer(34) | < 10^-6^ | 21.6% (0-67%) | 1.37-5.77 | 0.07 | 5/0.96 | 4.71×10^-6^ | Yes | Yes/Yes | Weak |
| Gastric cancer(36) | < 0.001 but >10^-6^ | 42.7% (0-83%) | 0.04-117.14 | 0.05 | 3/0.76 | 2.86×10^-3^ | Yes | Yes/Yes | Weak |
| Gastric cancer(36)** | < 0.05 but > 0.001 | 84.3% (53%-95%) | 0.03-80.28 | 0.37 | 2/1.05 | 0.25 | Yes | No/No | Weak |
| Gynecological cancers(30) | < 0.001 but > 10^-6^ | 85.8% (79%-90%) | 0.83-2.28 | 0.02 | 12/10.43 | 0.45 | No | Yes/No | Suggestive |
| Hormone-dependent cancer(24) | < 0.001 but > 10^-6^ | 70.8% (50%-83%) | 0.88-1.67 | 0.01 | 8/6.58 | 0.45 | No | Yes/No | Suggestive |
| Larynx cancer(35) | > 0.05 | 85.6% (58%-95%) | 0.00-90809.77 | 0.07 | 2/1.08 | 0.27 | Yes | Yes/No | No association |
| Lung cancer(34) | < 0.05 but > 0.001 | 38.6% (0-76%) | 0.83-2.95 | 0.00 | 5/1.38 | 4.49×10^-4^ | Yes | Yes/Yes | Weak |
| Oral cavity cancer(35) | < 10^-6^ | 0 (0-90%) | 0.44-11.33 | 0.30 | 3/0.22 | 1.03×10^-9^ | Yes | No/Yes | Weak |
| Ovarian cancer(30) | < 0.001 but > 10^-6^ | 0 (0-85%) | 1.01-1.98 | 0.39 | 3/0.51 | 1.85×10^-4^ | Yes | No/Yes | Suggestive |
| Overall cancer(34) | < 10^-6^ | 75.2% (67%-81%) | 1.01-2.48 | 0.00 | 38/19.52 | 2.03×10^-8^ | Yes | Yes/Yes | Highly suggestive |
| Overall cancer(34)** | < 10^-6^ | 77.7% (69%-84%) | 0.99-1.29 | 0.00 | 26/13.45 | 4.08×10^-6^ | Yes | Yes/Yes | Highly suggestive |
| Pharynx cancer(35) | < 10^-6^ | 20.3% (0-64%) | 1.17-3.48 | 0.30 | 4/1.52 | 0.01 | Yes | No/Yes | Highly suggestive |
| Prostate cancer(32) | < 0.001 but > 10^-6^ | 78.2% (60%-88%) | 0.80-3.74 | 0.00 | 8/5.17 | 0.07 | No | Yes/Yes | Suggestive |
| Prostate cancer(32)** | < 0.05 but > 0.001 | 72.9% (49%-86%) | 0.92-1.30 | 0.02 | 5/4.18 | 0.60 | No | Yes/No | Weak |
| Rectal cancer(25) | < 0.001 but > 10^-6^ | 54.5% (0-81%) | 0.93-2.24 | 0.85 | 4/1.73 | 0.05 | Yes | No/Yes | Suggestive |
| Respiratory tract cancer(24) | < 0.05 but > 0.001 | 69.1% (11%-89%) | 0.33-9.72 | 0.45 | 2/1.29 | 0.45 | No | No/No | Weak |
| UADT cancer(35) | < 10^-6^ | 60.2% (33%-76%) | 1.24-4.18 | 0.03 | 14/6.33 | 2.19×10^-8^ | Yes | Yes/Yes | Highly suggestive |
| **Mortality** | | | | | | | | | |
| All-cause mortality(29) | < 0.001 but > 10^-6^ | 72.6% (37%-88%) | 0.87-1.68 | 0.11 | 4/2.44 | 0.19 | Yes | No/No | Suggestive |
| Cancer mortality(24) | < 0.05 but > 0.001 | 54.1% (9%-77%) | 0.83-1.82 | 0.16 | 4/3.26 | 0.63 | No | No/No | Weak |
| CVD mortality(27) | < 10^-6^ | 70.8% (46%-84%) | 0.97-1.75 | 0.02 | 9/5.15 | 0.01 | Yes | Yes/Yes | Highly suggestive |
| CVD mortality(9)** | < 0.05 but > 0.001 | 76.3% (47%-89%) | 0.92-1.28 | 0.17 | 4/1.65 | 0.03 | Yes | No/Yes | Weak |
| **Metabolic outcomes** | | | | | | | | | |
| Central obesity(38) | > 0.05 | 65.4% (38%-81%) | 0.60-2.24 | 0.35 | 2/3.64 | 0.28 | No | No/Yes | No association |
| Hypertension(33) | < 0.05 but > 0.001 | 55.6% (21%-75%) | 0.80-1.60 | 0.55 | 6/3.80 | 0.17 | Yes | No/No | Weak |
| Hyperglycaemia(33) | > 0.05 | 60.7% (24%-80%) | 0.67-1.91 | 0.02 | 4/3.90 | 0.95 | No | Yes/No | No association |
| Metabolic syndrome(29) | > 0.05 | 32.6% (0-74%) | 0.58-1.74 | 0.70 | 1/0.95 | 0.95 | No | No/No | No association |
| **Other outcomes** | | | | | | | | | |
| CVD(27) | < 0.05 but > 0.001 | 37% (0-75%) | 0.78-2.54 | 0.03 | 3/2.20 | 0.50 | No | Yes/No | Weak |
| CVD(9)** | < 0.05 but > 0.001 | 71.2% (18%-90%) | 0.79-1.46 | 0.13 | 3/1.40 | 0.09 | Yes | No/Yes | Weak |
| Depression(28) | < 0.001 but > 10^-6^ | 0 (0-75%) | 1.08-1.41 | 0.99 | 3/0.53 | 4.06×10^-4^ | Yes | No/Yes | Suggestive |

Abbreviation：CVD: cardiovascular disease; DII: dietary inflammatory index; UADT: upper aerodigestive tract.

§ Expected number of statistically significant studies using the point estimate of the largest study (smallest standard error) as the plausible effect size.

# Observed/Expected number of statistically significant studies

¶ *P* value of the excess statistical significance test.

* *P* value under the random-effects model.

** Exposure as continuous variable.

**Supplementary Table S6. Sensitivity analysis limited to prospective cohort studies for associations with convincing and highly suggestive evidence in the main analysis**

| Outcomes (reference) | No. of studies | Significance threshold reached* | Number of cases/controls | Largest study significant | 95% PI | I^2^ (95% CI) | Small-study effects/Excess statistical significance | Evidence class |
| --- | --- | --- | --- | --- | --- | --- | --- | --- |
| Colorectal cancer**^31^ | 4 | 9.57×10^-32^ | 14888/867624 | Yes | 1.02-1.05 | 0 (0-85%) | Yes/Yes | highly suggestive |
| CVD mortality^27^ | 10 | 4.09×10^-8^ | 32319/385765 | Yes | 0.97-1.75 | 70.8% (46%-84%) | No/Yes | highly suggestive |
| Digestive tract  Cancer^24^ | 3 | 2.36×10^-6^ | 9781/676681 | Yes | 0.43-3.86 | 47.5% (0-85%) | No/Yes | suggestive |
| Overall cancer^34^ | 16 | 1.75×10^-9^ | 29606/1246387 | Yes | 1.00-1.67 | 58.3% (27%-76%) | Yes/Yes | highly suggestive |
| Overall cancer**^34^ | 9 | 4.12×10^-4^ | 19058/497780 | Yes | 0.94-1.26 | 80.6% (64%-90%) | Yes/Yes | suggestive |

CVD: cardiovascular disease; PI: prediction interval.

* *P* value under the random-effects model.

** Exposure as continuous variable.


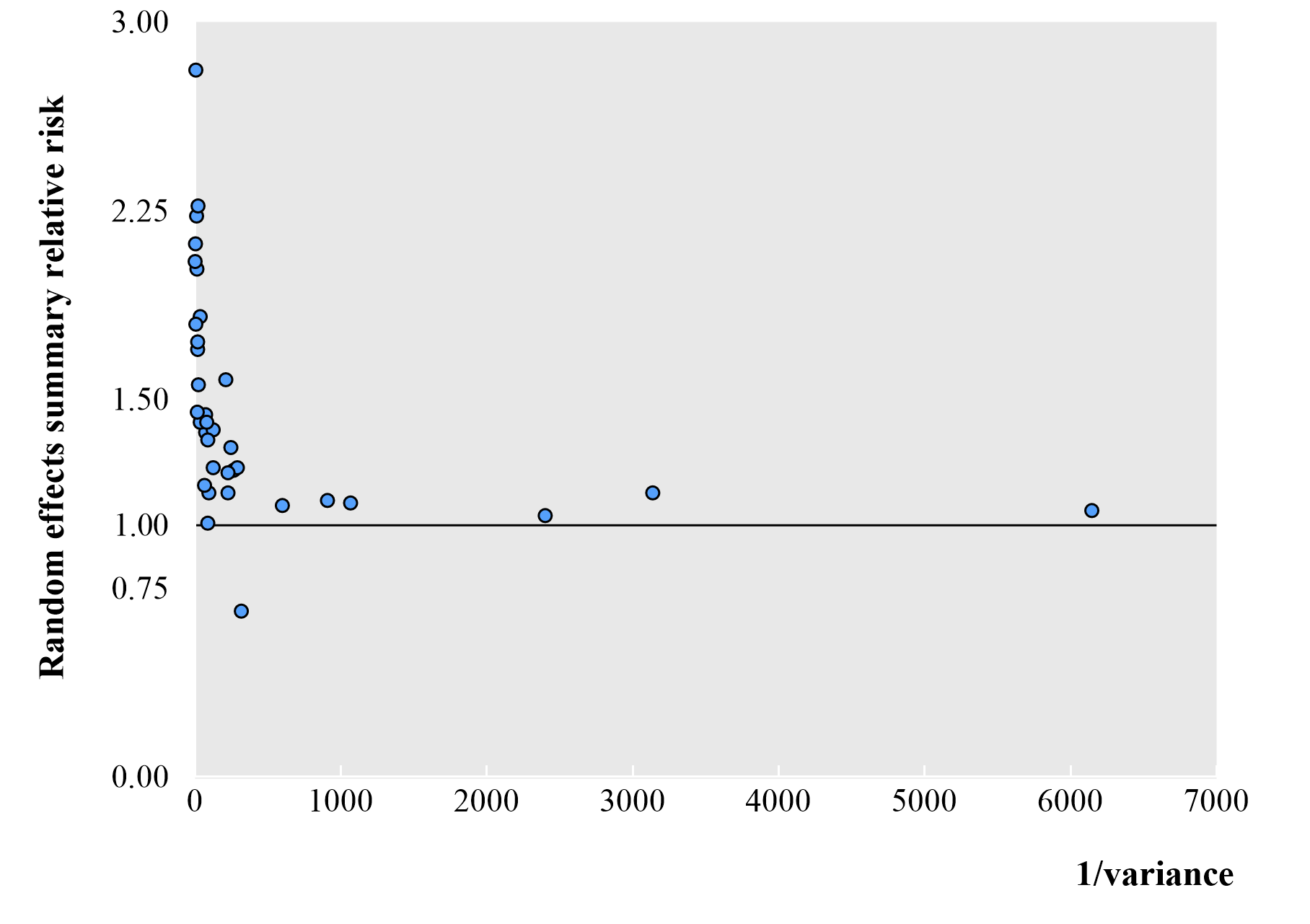


**Supplementary Figure 1. Association of meta-analysis summary effects sizes with inverse of the variance.**

**
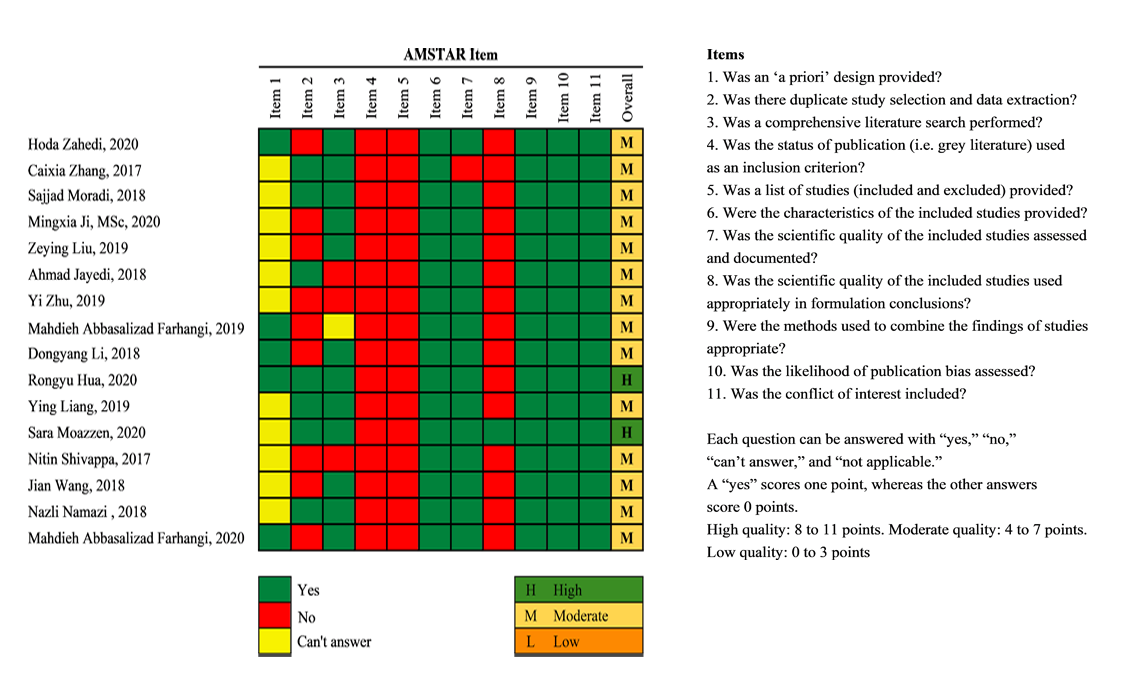
**

**Supplementary Figure 2 Detailed evaluation of the methodological quality with AMSTAR**
